# Ministernotomy in Aortic Root and Arch Surgery: Early Outcomes

**DOI:** 10.21470/1678-9741-2021-0372

**Published:** 2023

**Authors:** Ulku Kafa Kulacoglu, Mehmet Kaya

**Affiliations:** 1 Department of Cardiovascular Surgery, Republic of Turkey Ministry of Health, University of Health Sciences Istanbul Mehmet Akif Ersoy Thoracic and Cardiovascular Surgery Training and Research Hospital.

**Keywords:** Sternotomy, Thoracic Surgery, Aorta, Thoracic, Erythrocytes, Morbidity

## Abstract

**Introduction:**

Minimally invasive methods have become more preferred in cardiac surgery today. In this study, the comparative results of patients who underwent an aortic root, arch or hemiarch replacement by ministernotomy and full sternotomy in our clinic are presented.

**Methods:**

Between January 2017 and October 2019, a series of operations including aortic root, ascending aorta, and aortic arch replacements were performed on 278 patients. The ministernotomy technique was used in 25 of them. Twenty patients who underwent full sternotomy were selected and matched to this group for comparison.

**Results:**

The ministernotomy group had a longer cross-clamping time (128.3±30.8 *vs.* 104.7±23.4 min, *P*=0.007) but the total operating time was similar in the two groups (249.76±28.56 *vs.* 248.25±37.53 min, *P*=0.879). The number of red blood cell (RBC) transfusions per patient was higher in the full sternotomy group (4.65±3.74 *vs.* 2.44±1.85 unit, *P*=0.020). The ministernotomy group had shorter ventilation times (7.60±4.88 *vs.* 32.30±32.25 h, *P*<0.001) and shorter ICU stay (1.56±0.58 *vs.* 3.35±1.46 d, *P*<0.001). The 30-day mortality was 0% in the ministernotomy group.

**Conclusion:**

Early results of our study show that, in combined or isolated aortic root, ascending aorta, and aortic arch surgeries, ministernotomy can be applied with relatively safety and low mortality and morbidity rates.

## INTRODUCTION

Minimally invasive cardiac surgery is becoming increasingly preferred due to its rapid recovery process, shorter hospital and intensive care unit (ICU) stays, reduced postoperative pain, and good cosmetic results. Holman and Willett reported the first approach using partial sternotomy in the treatment of pericardiectomy in 1949^[1]^. In addition to its cosmetic advantage, limited bleeding, rapid extubating, and brevity of ICU and hospital stays are just a few of the advantages of partial sternotomy^[2,3,4]^. As stated in many studies, single procedures such as aortic valve replacement (AVR) operations with ministernotomy have been performed routinely in many centers for some time with good results^[5,6]^. However, today, complex procedures such as aortic root and aortic arch operations are still performed in many centers with classic median sternotomy. The aim of this study is to demonstrate the safe and effective applicability of aortic root and aortic arch replacement surgeries with ministernotomy. In our single-center and retrospective study, the early results of 45 patients who underwent aortic root, ascending aorta, and aortic arch replacement with upper J-shaped ministernotomy and full sternotomy methods were compared and presented for ascending and aortic arch aneurysm and aortic valve disorders.

## METHODS

Between January 2017 and October 2019, 278 patients aged 24 to 77 (54.29±13.98) at our clinic underwent operations involving aortic root, ascending aorta, and aortic arch replacements for aortic disorders. The mean logistic EuroSCORE was 1.79±0.17. The ministernotomy technique was used in 25 of the 278 patients. Twenty patients who underwent full sternotomy with similar demographic characteristics such as age, gender, and logistic EuroSCORE and were operated on by the same surgical team were selected and matched with this group for comparison. The preoperative demographic data of the patients from each group are shown in Table 1. There was no statistically significant diference between the two groups in terms of comorbidity and demographic data. The way the surgeries would be performed was decided by the cardiovascular team with support from the board. Emergency cases were excluded. Case parameters before, during, and after surgery were analyzed retrospectively.

**Table 1:** Preoperative demographic data and comorbidities.

	Upper ministernotomy (n=25)	Full sternotomy (n=20)	*P*-value
Age	54.88±13.20	53.55±15.21	0.755
Males	17 (68.0)	18 (90.0)	0.147
BSA	1.92±0.17	1.96±0.16	0.409
Diabetes mellitus	3 (12.0)	5 (25.0)	0.435
Dyslipidemia	9(36.0)	6 (30.0)	0.671
HT	10 (40.0)	7 (35.0)	0.731
Smoking	9 (36.0)	9 (45.0)	0.540
COPD	7 (28.0)	3 (15.0)	0.473
Renal insufficiency	2 (8.0)	0	0.495
Stroke	0	1 (5.0)	0.444
CAD	2 (8.0)	3 (15.0)	0.642
Bicuspid aortic valve	6 (24.0)	6 (30.0)	0.651
Logistic EuroSCORE	2.21±1.51	1.26±0.33	0.080

BSA=body surface area; CAD=coronary artery disease; COPD=chronic obstructive pulmonary disease; HT=hypertension. Note: data presented as mean±SD (standard deviation) or n (%).

**Fig. 1:**
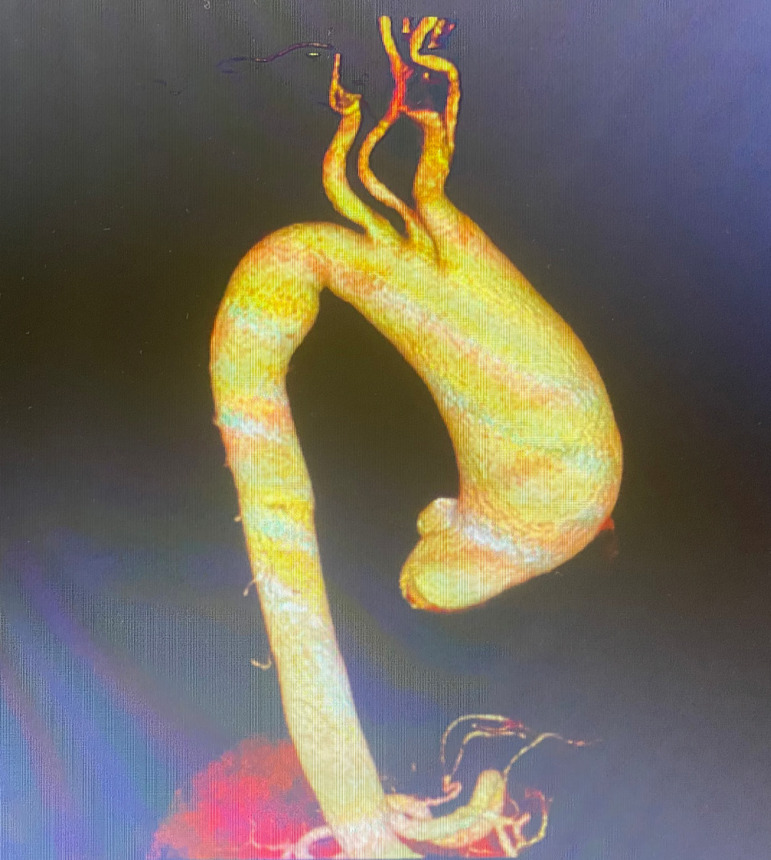
Preoperative 3D computed tomography image of a patient with ascending and aortic arch aneurysm.

All patients underwent preoperative transthoracic echocardiography, coronary angiography, and contrast-enhanced computed tomography (CT) to determine the diameter and configuration of the aorta (Figure 1). Approval was obtained from the institutional ethics committee (2020-06).

### Ministernotomy Technique

All patients were placed in the supine position. External defibrillator pads were placed on the patient’s chest. The incision site marking was performed with a marker. We made a skin incision starting at the sternal angle and extending to the 3^rd^ or 4^th^ intercostal space. We applied upper J-shaped ministernotomy through the 3^rd^ or 4^th^ intercostal space according to the location of the ascending aorta on contrast-enhanced CT.

We used the De Soutter Medical MBC sternudrive lite™ for sternotomy. There was no need for ligation of the internal thoracic artery in any patient. This incision provided a satisfactory exposure for central venous and arterial cannulation. The average incision size was 7±1 cm. We removed the thymus and mediastinal adipose tissue. In patients who underwent hemiarch and total aortic arch replacement, we mobilized and tractioned the innominate vein. We opened the pericardium longitudinally and hung it with three traction stitches on the right and left sides. We reinserted a minimally invasive sternum retractor. Thus, we achieved maximum surgical exposure by anteriorizing the ascending aorta. We performed venous cannulation in all patients from the right atrium. For this, we used FreeLife™ Medical GmbH two-stage venous cannula fatbody. Patients with femoral and jugular vein cannulation were excluded from the study. Eighteen patients (72%) who underwent Bentall and ascending aorta operations underwent 28°C mild hypothermia. In these patients, cardiopulmonary bypass (CPB) was accomplished using the total central cannulation technique. In 7 patients (28%) who underwent hemiarch and total arch replacement operations, total circulatory arrest was achieved by cooling the patient’s body temperature to 20°C. In these patients, we performed right axillary artery and right atrium cannulation. We achieved antegrade cerebral perfusion by brachiocephalic artery and left carotid artery cannulation. In all patients, the left ventricle was vented by placing a vent in the right pulmonary vein. We achieved myocardial protection by delivering 6°C of cold blood cardioplegia every 15 minutes from the coronary ostia selectively. We used CO_2_ to prevent air embolism. We resected the aneurysmatic aortic tissue after aortotomy. In patients undergoing the Bentall procedure, buttons were prepared by dissecting the coronary ostia. We used valved conduits that were prepared with Mitroflow™ biological valves and Vascutek™ Valsalva grafts in 3 patients over 70 years old who needed biological conduits for Bentall operations. We used the previously described “French cuf” technique in all patients undergoing a Bentall operation^[7]^. In patients who underwent total arch and hemiarch replacement, we used two separate grafts and then sutured them together.

After the aortotomy was closed, we placed two temporary pacing wires on the right ventricle and a chest tube (32 F) from the subxiphoid region for later fixation. We removed the cross-clamp after deairing. In addition, all patients underwent intraoperative transesophageal echocardiography for control. We closed the sternum with four separate sternal wires.

### Statistical Analysis

Data obtained in the research were evaluated with the Statistical Package for the Social Sciences version 21 software (IBM SPSS Statistics, Armonk, NY, USA). In descriptive statistics, categorical variables are presented as numbers and percentages, and numerical variables are presented as mean, standard deviation, and median minimum and maximum values using interquartile range. The conformity of the numerical variables with the normal distribution was evaluated with the Shapiro-Wilk test. In the comparisons of two independent groups, the t-test was used when parametric conditions were met and the Mann-Whitney U test when parametric conditions were not met. Chi-square and Fisher’s exact tests were used in the analysis of categorical variables. Cases where type I error was below 5% (*P*<0.05) were considered statistically significant.

## RESULTS

**Fig. 2:**
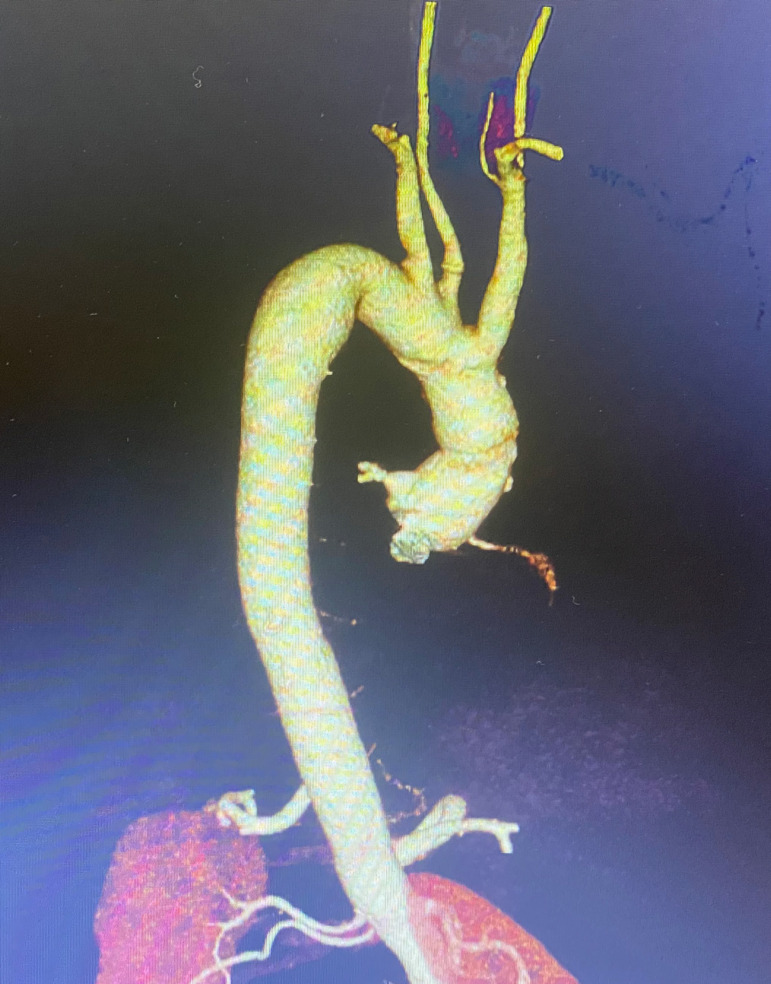
Postoperative 3D computed tomography image of the patient after total arch replacement.

Intraoperative data for both groups and operative procedures performed (ascending aorta and aortic arch surgery combined or not combined with valve or root) are shown in Table 2. In the ministernotomy group, 25 patients were operated on. The Bentall operation was performed in 21 patients (one of whom had a redo Bentall operation), simultaneous hemiarch replacement in 4 patients and simultaneous total arch replacement in 2 patients. Another patient underwent ascending aortic replacement due to chronic dissection. In 3 patients over 70 years old, we performed the Bentall procedure with a composite biological conduit made with a Mitroflow biological valve and a Jotec Dacron graft. We used a mechanical valve conduit with sinus of Valsalva in Bentall procedures in 21 patients under 70 years old. In 2 patients who underwent total arch replacement, the supra-aortic vessels were reimplanted as an island. A postoperative CT image is provided in Figure 2. In the full sternotomy group, the types of surgeries performed on the 20 patients included in the study were almost similar to the ministernotomy group. Seventeen patients underwent Bentall operations; in addition, hemiarch replacement was performed in 2 patients and total arch replacement in 1 patient.

**Table 2:** Intraoperative variables.

	Upper ministernotomy (n=25)	Full sternotomy (n=20)	*P*-value
Type of surgery			0.259
Root + AA	14 (56.0)	14 (70.0)	
Root + AA + hemiarch	4 (16.0)	2 (10.0)	
Root +AA+ total arch	2 (8.0)	1 (5.0)	
AV + AA	2 (8.0)	1 (5.0)	
AV + AA + hemiarch	1 (4.0)	0	
AA	1 (4.0)	2 (10.0)	
Redo root + AA	1 (4.0)	0	
CPB time (min)	194.28±28.44	147.65±31.18	<0.001
Cross-clamp time (min)	128.28±30.82	104.75±23.42	0.007
Operating time (min)	249.76±28.56	248.25±37.53	0.879
HCA	7 (28.0)	4 (20.0)	0.729

AA=ascending aorta; AV=aortic valve; CPB=cardiopulmonary bypass; HCA=hypothermic circulatory arrest. Note: data presented as mean±SD (standard deviation) or n (%).

### Surgical Outcomes

When we compared the groups in terms of CPB duration, we found this to be significantly longer in the ministernotomy group (147.65±31.18 *vs.* 194.28±28.44 *P*<0.001). The mean cross-clamp time in the classical sternotomy group was 104.7±23.4 and in the ministernotomy group it was 128.3±30.8. As a result of the analysis, the diference observed between the groups was statistically significant (*P*=0.007). There was no significant diference between the groups in terms of hypothermic circulatory arrest time and operative time (*P*=0.19, *P*=0.87). Postoperative results are listed in Table 3. Only 1 patient out of 45 patients (2.2%) in the full sternotomy group had died. There was no statistically significant diference between the groups (*P*=0.44).

**Table 3:** Postoperative data.

	Upper ministernotomy (n=25)	Full sternotomy (n=20)	*P*-value
RBC transfusion (U)	2.44±1.85	4.65±3.74	0.020
FFP transfusion (U)	4.80±3.62	7.65±5.22	0.038
Ventilation time (hours)	7.60±4.88	32.30±32.25	<0.001
ICU stay (days)	1.56±0.58	3.35±1.46	<0.001
Hospital stay (days)	12.76±7.01	14.45±5.59	0.199
Renal failure (new occurrence)	4 (16.0)	4 (20.0)	1.000
Neurological complications	0	1 (5.0)	0.444
Rethoracotomy	2 (8.0)	7 (35)	0.057
Mediastinitis	1 (4.0)	2 (10.0)	0.577
Mortality (30 days)	0	1 (5.0)	0.444

FFP=fresh frozen plasma; ICU=intensive care unit; RBC=red blood cells. Note: data presented as mean±SD (standard deviation) or n (%).

When the groups were compared in terms of the number of red blood cell (RBC) and fresh frozen plasma (FFP) transfusions, the use of blood products was significantly less common in the ministernotomy group. In the classic sternotomy group, the mean number of RBC units used was 4.65±3.74 and the mean number of RBC units used in the ministernotomy group was 2.44±1.85. As a result of the analysis, the diference observed between both groups was statistically significant (*P*=0.02). While the mean use of FFP was 7.65±5.22 in the full sternotomy group, the mean use was 4.80±3.62 in the ministernotomy group. This is also statistically significant (*P*=0.038).

When mechanical ventilation time (7.60±4.88 *vs.* 32.30±32.25; *P*<0.001) and ICU stay (1.56±0.58 *vs.* 3.35±1.46; *P*<0.01) were compared in patients who underwent full sternotomy and ministernotomy, ministernotomy patients’ statistics were significantly lower. The length of hospital stay was 12.76±7.01 days for ministernotomy, shorter than full sternotomy (14.45±5.59). However, this is not a statistically significant diference (*P*=0.19). Rethoracotomy rates in ministernotomy were lower (*P*=0.57). When we consider the rates of mediastinitis, the results of the two groups were similar.

Postoperative renal failure and rates of neurological events were similar in both groups. There was no need for postoperative dialysis in either group. No conversion to full sternotomy was required in any patient. Reoperation was performed in 1 patient due to bleeding and in another patient due to pericardial tamponade in the ministernotomy group.

## DISCUSSION

Bentall and aortic arch operations have been safely performed with full median sternotomy for many years. However, some of the disadvantages are patients feeling intense pain even when breathing and therefore not being mobilized early, abnormal wound healing due to sternal instability and the occurrence of undesirable conditions such as mediastinitis. With the development of surgery, minimally invasive methods are gradually increasing. In particular, isolated AVR operations are routinely performed in many clinics worldwide with J-shaped sternotomy, which is a less invasive method. Evolving technology has provided various instruments that facilitate minimally invasive methods^[8]^. Compared with full sternotomy, the advantages of ministernotomy include reduced need for blood transfusion due to low bleeding, low mechanical ventilator requirements, short ICU and hospital stays, and low risk of postoperative soft tissue infection and mediastinitis^[2]^. Patients return to their normal lives after a short period of time. In addition, if they need a second heart surgery in the future, the risk of having to redo the surgery is slightly reduced because there will be less adhesion in the heart compared to a full sternotomy^[9]^. A variety of methods have been applied in ministernotomy: J-shaped sternotomy, L-shaped hemisternotomy, and T-shaped sternotomy. We found the best method to reach the ascending aorta and aortic root with J-shaped sternotomy. We do not find T-shaped sternotomy suitable because it causes sternal instability.

In a limited number of centers, Bentall operations with minimally invasive methods are performed with aortic arch replacement, and the studies performed on this are very limited^[3,10,11,12,13]^. Tabata et al.^[14]^ compared 79 patients in two groups (*i.e.*, classical full sternotomy and ministernotomy). However, none of these patients received total arch replacement. In general, there was no diference in mortality and morbidity. However, there was a significant diference in the amount of bleeding and length of ICU and hospital stays.

Today, studies show that not only isolated AVR but even complex operations such as aortic root and Ross procedures can be safely performed with ministernotomy^[15,16]^. Totaro et al.^[17]^ presented the largest series (1,126 cases, 67 Bentall) including upper hemisternotomy and concomitant procedures. The largest study series, which focused on aortic arch replacement with a minimally invasive method, was conducted by Goebel et al.^[18]^ with 21 patients. Its mortality and morbidity rates are very low.

While this method has only been used in selected cases in the past, it can now be safely applied to all kinds of complex cases, including redo cases, just like in our series. In our clinic, we have routinely using the upper J-shaped ministernotomy technique in uncomplicated isolated AVR cases for approximately 10 years. We developed this technique over time and started to apply it in complex cases. We routinely performed these surgeries with peripheral cannulation during the learning curve process. With the increase in our surgical experience, now we use the central cannulation technique in all cases that do not require selective cerebral perfusion.

In this study, we compared the results of patients who underwent non-emergency aortic root and ascending aortic surgery with ministernotomy and full sternotomy. We observed that there was no significant diference in the early period in terms of postoperative renal failure and cerebral complications. However, there was a significant diference in favor of ministernotomy in terms of early respiratory recovery and short ICU stay. CPB and cross-clamp times were significantly longer in ministernotomy. However, the total operating times were similar in both groups. The length of hospital stay was shorter in ministernotomy, but it was not statistically significant. The use of blood products was higher in full sternotomy, but we attribute this to the greater bleeding in this procedure. Bleeding is the biggest problem in aortic root repairs. We minimized bleeding using the French cuf technique (Video 1).

**Video 1:**
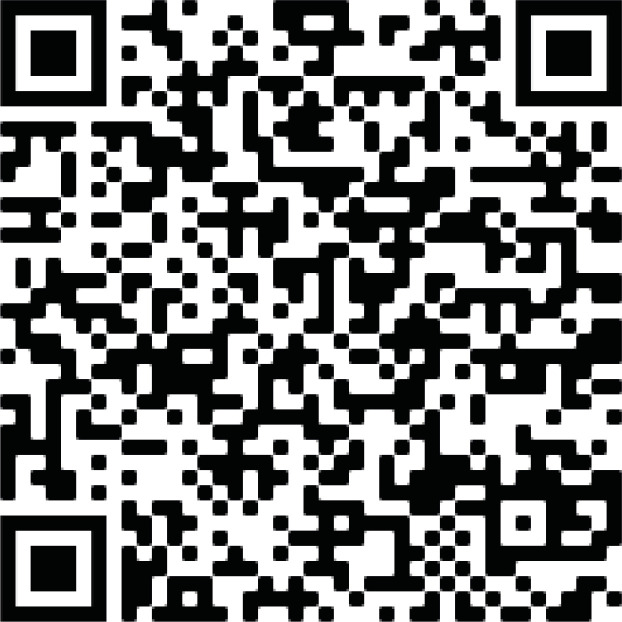
French Cuf technique, to ensure proximal annular hemostasis.

The reason we prefer central cannulation in most cases is that peripheral cannulation is also a rather invasive procedure. We used the right axillary artery for cannulation since we performed arch surgery in only 7 (28%) patients, which allowed us to perform cerebral perfusion during deep circulatory arrest.

Regardless of the diameter of the aneurysm, a large area remains for the surgeon to work in when the aneurysmal tissue is removed. For this reason, the large size of the aneurysm did not limit us in performing this procedure. The superiority of the ministernotomy compared to another minimally invasive method, the minithoracotomy, is that it can be easily converted to a full sternotomy when the patient’s safety is compromised. Although we have never needed this in our limited case series, we predict that as the number of cases increases, our rate of return to full sternotomy (0%) and mortality rate (0%) will increase.

Compared to the 4% stroke rate in the literature, the 0% rate in the ministernotomy group is quite low. The blood usage rate was slightly higher than what the literature indicates, which is 1.6 to 2%, with an average of 2.4 packed RBC units in the ministernotomy group. We expect our blood usage to decrease as our experience increases. Our revision rate is one (4%) due to tamponade in the ministernotomy group. This occurs because, due to limited pericardiotomy, the risk of tamponade is higher in partial sternotomies compared to full sternotomies. Our cross-clamp and CPB times in the ministernotomy group were somewhat longer compared to studies with large case series in the literature. We expect our operation times to decrease as our number of cases increases.

There are some disadvantages in addition to the advantages of ministernotomy. For example, sometimes unintentionally, internal thoracic artery injuries and consequent ligation requirements may arise. In addition, it can be quite difficult to deal with small incisions and limited exposure in complications developing intraoperatively. For these reasons, the success of the technique is proportional to the experience of the surgeon. In our study, none of the patients who underwent ministernotomy needed to switch to full sternotomy. The low rates of postoperative major complications, early respiratory recovery and short ICU stay, and low blood usage indicate that this technique is a viable and safe option.

### Limitations

The limitations of our study included that it is a single-center and not a multicenter study; hence, a small number of cases were studied and there was a lack of long-term result. In addition, upper J-shaped ministernotomy is not easily applicable in emergency surgeries and infectious endocarditis cases. We recommend applying this surgical technique in multiple centers considering more applicable cases to obtain long-term results and conclusions about the use of upper J-shaped ministernotomy. Furthermore, a prospective randomized study will help in the judgement between pros and cons of ministernotomy.

## CONCLUSION

Complex surgeries such as aortic root, ascending aorta, and aortic arch surgeries can be safely performed with the upper J-shaped ministernotomy technique with low mortality and morbidity. In addition, short ICU stay, less use of blood products, faster recovery, and better cosmetic results are among the advantages. Ministernotomy might be the first choice approach in elderly patients, especially those with smoking history and who had been diagnosed with chronic obstructive pulmonary disease. However, more case series are needed in this field. We hope that our study will encourage other surgical teams to use this minimally invasive method.
